# Management of TAVI Candidates With Metal Allergy: Minimally Invasive Aortic Valve Replacement With a Metal-Free Bioprosthesis

**DOI:** 10.1016/j.atssr.2025.10.015

**Published:** 2025-11-19

**Authors:** Naoto Tanabe, Hiroshi Tsuneyoshi, Tomofumi Taki, Shoichi Kyo, Akira Takeuchi, Koki Tamaoka, Ryo Nakanishi, Hiroto Kawakami

**Affiliations:** Department of Cardiovascular Surgery, Shizuoka General Hospital, Shizuoka, Japan

## Abstract

An 87-year-old woman with severe aortic stenosis required intervention. Patch testing demonstrated nickel and cobalt hypersensitivity, ruling out transcatheter valve implantation. After multidisciplinary review, minimally invasive aortic valve replacement through a right anterior thoracotomy was chosen, thereby avoiding median sternotomy and stainless steel wires. A 21-mm Avalus bioprosthesis (Medtronic)—engineered without a metallic stent—was implanted successfully. Recovery was smooth. Extubation occurred within hours, and the patient was discharged from the hospital on postoperative day 5. At 1 year, prosthetic performance and clinical status were excellent. This experience illustrates a practical path when transcatheter treatment is contraindicated by documented metal allergy: minimally invasive surgery with a metal-free valve.

Metal hypersensitivity, although uncommon, represents a clinically relevant challenge in contemporary valve therapy because most transcatheter and surgical prostheses incorporate metallic elements such as nickel, cobalt, and chromium.^1^ Notably, patients with proven metal allergies may experience allergic reactions or complications from the implanted devices, and transcatheter aortic valve implantation (TAVI) is often contraindicated in these situations. Consequently, surgical alternatives remain essential, particularly when combined with minimally invasive cardiac surgery (MICS), to reduce surgical morbidity.Recent advances in prosthetic designs have facilitated the development of metal-free bioprostheses. Importantly, an Avalus (Medtronic) valve lacking metallic stent components was successfully implanted in patients with suspected metal hypersensitivity.^2^ Careful preoperative evaluation, including dermatologic consultation, is crucial for selecting appropriate prosthetic materials and minimizing the risk.^3^

An 87-year-old woman with a history of hypertension, type 2 diabetes mellitus, osteoporosis, and dyslipidemia presented with exertional dyspnea corresponding to New York Heart Association functional class II exertional dyspnea. Transthoracic echocardiography (TTE) at the referral hospital revealed severe aortic stenosis, and she was referred to our institution (Shizuoka General Hospital, Shizuoka, Japan) for consideration of TAVI. During the preoperative evaluation, the patient reported a history of metal allergy. A dermatologic consultation was obtained, and patch testing was performed using 17 metals and titanium applied to the patient’s back. One week later, erythematous reactions were observed, confirming nickel and cobalt hypersensitivity. Therefore, TAVI was considered contraindicated. After a multidisciplinary discussion, the treatment strategy shifted to MICS aortic valve replacement (AVR).

On admission, her height, weight, and body surface area were 141 cm, 40 kg, and 1.25 m^2^, respectively. Physical examination revealed a palpable peripheral pulse and a grade III systolic murmur at the right sternal border. Laboratory test results demonstrated mild anemia and elevated brain natriuretic peptide. TTE exhibited a preserved left ventricular ejection fraction of 80%, a left ventricular end-diastolic/systolic diameter of 45/26 mm, a peak transaortic velocity of 4.1 m/s, and an aortic valve area of 0.63 cm^2^. The mean stroke volume index was 41.6 mL/m^2^. The calculated European System for Cardiac Operative Risk Evaluation II (EuroSCORE II) was 2.17, and The Society of Thoracic Surgeons predicted mortality risk was 13.6%. Contrast-enhanced computed tomography revealed mild calcification of the ascending aorta and suitable peripheral access vessels. Coronary angiography demonstrated no significant stenosis.

The patient underwent MICS AVR through a 5-cm right anterior thoracotomy (RAT) in the third intercostal space (RAT approach). Cardiopulmonary bypass was performed through the right femoral artery and vein. A 21-mm Avalus bioprosthesis was successfully implanted. The total operative, cardiopulmonary bypass, and aortic cross-clamp times were 206, 121, and 79 minutes, respectively. The estimated blood loss was 200 mL.

The patient was extubated 4 hours postoperatively and was transferred to the general ward on postoperative day (POD) 1. The thoracic drain was removed on POD 2, and the patient was discharged on POD 5, with an uneventful recovery. Early postoperative TTE demonstrated stable prosthetic valve function with a peak velocity, mean pressure gradient, and aortic valve area of 2.3 m/s, 10 mm Hg, and 1.68 cm^2^, respectively. At the latest follow-up, approximately 1 year postoperatively, the patient remained in good health, with satisfactory functional status.

## Comment

Although uncommon, metal hypersensitivity is a clinically relevant challenge in cardiovascular surgery. Dermal metal allergy is reported in 10% to 15% of the general population, whereas implant-related reactions are rare (<0.1%) but may cause serious complications such as systemic inflammation, prosthetic dysfunction, and device failure.^1^^,^^4^ The underlying mechanism involves corrosion and the subsequent release of metal ions, particularly nickel, cobalt, and chromium, which can trigger T-cell–mediated immune responses and delayed hypersensitivity reactions.^5^ Moreover, in cardiovascular practice, hypersensitivity has been linked to in-stent restenosis and bioprosthetic valve dysfunction through endothelial injury and inflammatory activation.^2^^,^^6^

Our patient demonstrated hypersensitivity to nickel and cobalt during patch testing. This finding is clinically significant because most commercially available transcatheter aortic valve systems incorporate metallic stent frames composed of nickel, cobalt, or chromium alloys, which are recognized allergens in sensitized patients. Therefore, TAVI was contraindicated in this case. Similarly, most surgical bioprosthetic valves contain metallic components in their structures. However, the Avalus valve is designed without a metal stent frame, thus making it a suitable option for patients with proven metal hypersensitivity. Previous reports have documented the successful use of this valve in patients with suspected or confirmed metal allergies, thereby further supporting its applicability.^2^

Chest closure is an important consideration in surgical planning. Median sternotomy with stainless steel wires was avoided because such wires typically contain nickel, and hypersensitivity reactions to sternal wires has been associated with systemic inflammation, persistent pain, and pruritus, often resolving only after wire removal.^7^^,^^8^ Therefore, conventional median sternotomy was deemed inappropriate, and a minimally invasive approach was selected.

At our institution, MICS AVR is typically performed through either a RAT or thoracotomy in the second intercostal space (TAX). In TAX procedures, automated knotting devices such as the Cor-Knot device (LSI Solutions) are frequently required. Although the implant material of Cor-Knot is pure titanium—which is rarely associated with hypersensitivity in nickel- or cobalt-allergic patients—we aimed to minimize any unnecessary metallic material introduction. Furthermore, even though manual knot tying with a knot pusher allows TAX without the use of Cor-Knot, it prolongs the cardiopulmonary bypass time and increases the operative burden. Therefore, the RAT approach was selected to enable safe manual suture tying without using metallic adjuncts.

This case report highlights several important findings. First, preoperative screening for metal allergies, including detailed history taking and patch testing, is essential for patients undergoing cardiovascular interventions. Second, the development and clinical adoption of metal-free or surface-modified hypoallergenic devices provide critical options for managing sensitized patients. Finally, MICS AVR using a metal-free bioprosthesis represents a feasible and effective alternative for patients with contraindications to TAVI because of metal allergies, by offering both surgical safety and favorable postoperative recovery.

## Declaration of Generative AI and AI-Assisted Technologies in the Writing Process

During the preparation of this work the authors used ChatGPT for English proofreading. After using this tool/service, the authors reviewed and edited the content as needed and take full responsibility for the content of the published article.
